# Aberrantly elevated suprabasin in the bone marrow as a candidate biomarker of advanced disease state in myelodysplastic syndromes

**DOI:** 10.1002/1878-0261.12768

**Published:** 2020-08-11

**Authors:** Miroslav Pribyl, Sona Hubackova, Alena Moudra, Marketa Vancurova, Helena Polackova, Tomas Stopka, Anna Jonasova, Radka Bokorova, Ota Fuchs, Jan Stritesky, Barbora Salovska, Jiri Bartek, Zdenek Hodny

**Affiliations:** ^1^ Department of Genome Integrity Institute of Molecular Genetics of the Czech Academy of Sciences Prague Czech Republic; ^2^ Molecular Therapy of Cancer Group Institute of Biotechnology of the Czech Academy of Sciences BIOCEV Prague Czech Republic; ^3^ 1st Department of Medicine First Faculty of Medicine Charles University in Prague and General University Hospital Prague Czech Republic; ^4^ Group of Mechanisms Involved in Remodeling of Chromatin Structure During Cell Fate Decisions BIOCEV Prague Czech Republic; ^5^ Department of Genomics Institute of Hematology and Blood Transfusion Prague Czech Republic; ^6^ Institute of Pathology First Faculty of Medicine Charles University and General University Hospital in Prague Prague Czech Republic; ^7^ Danish Cancer Society Research Center Copenhagen Denmark; ^8^ Division of Genome Biology Department of Medical Biochemistry and Biophysics Science for Life Laboratory Karolinska Institute Stockholm Sweden

**Keywords:** 5‐azacytidine, biomarker, MDS, MDSCs, suprabasin

## Abstract

Myelodysplastic syndromes (MDS) are preleukemic disorders characterized by clonal growth of mutant hematopoietic stem and progenitor cells. MDS are associated with proinflammatory signaling, dysregulated immune response, and cell death in the bone marrow (BM). Aging, autoinflammation and autoimmunity are crucial features of disease progression, concordant with promoting growth of malignant clones and accumulation of mutations. *Suprabasin* (*SBSN*), a recently proposed proto‐oncogene of unknown function, physiologically expressed in stratified epithelia, is associated with poor prognosis of several human malignancies. Here, we showed that *SBSN* is expressed in the BM by myeloid cell subpopulations, including myeloid‐derived suppressor cells, and is secreted into BM plasma and peripheral blood of MDS patients. The highest expression of *SBSN* was present in a patient group with poor prognosis. *SBSN* levels in the BM correlated positively with blast percentage and negatively with CCL2 chemokine levels and lymphocyte count. *In vitro* treatment of leukemic cells with interferon‐gamma and demethylating agent 5‐azacytidine (5‐AC) induced *SBSN* expression. This indicated that aberrant cytokine levels in the BM and epigenetic landscape modifications in MDS patients may underlie ectopic expression of *SBSN*. Our findings suggest SBSN as a candidate biomarker of high‐risk MDS with a possible role in disease progression and therapy resistance.

## Graphical abstract

Aberrantly expressed *suprabasin* (*SBSN*) is a novel biomarker in myelodysplastic syndrome (MDS) patients. The highest levels of SBSN, secreted from myeloid subpopulations, including myeloid‐derived suppressor cells, are detectable within bone marrow (BM) and peripheral blood plasma of poor prognosis MDS subgroup. SBSN negatively correlated with BM T cells and CCL2 levels indicating immunosuppressive milieu in BM of MDS patients.

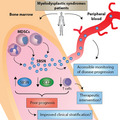

**Abbreviations**
BM: bone marrow
BV421: Brilliant Violet™ 421
CCL2: C‐C motif chemokine ligand 2
CD: cluster of differentiation
CO_2_: carbon dioxide
DPX: distyrene–plasticizer–xylene
EB‐1: excess blasts‐1
EB‐2: excess blasts‐2
FACS: fluorescence‐activated cell sorting
FSC: forward scatter
GAPDH: glyceraldehyde 3‐phosphate dehydrogenase
H_2_O_2_: hydrogen peroxide
HLA‐DR: human leukocyte antigen–DR isotype
HRP: horseradish peroxidase
IL: interleukin
int: intermediate
IP‐10: interferon‐gamma‐induced protein 10
IRF1: interferon regulatory factor 1
Lin: lineage
LRG1: leucine‐rich alpha‐2‐glycoprotein 1
MCF‐7: Michigan Cancer Foundation‐7
MDS: myelodysplastic syndromes
MDSC: myeloid‐derived suppressor cell
MLD: multilineage dysplasia
mRNA: messenger RNA
NaHCO_3_: sodium bicarbonate
NH_4_Cl: ammonium chloride
OCI‐M2: Ontario Cancer Institute M2
Opti‐MEM: minimal essential medium
pH: power of hydrogen
RNA: ribonucleic acid
RT: room temperature
SSC: side scatter
STAT1: signal transducer and activator of transcription 1
SV40: simian vacuolating virus 40
TGF‐β: transforming growth factor‐β
TNF‐α: tumor necrosis factor‐α

## 1 Introduction

Myelodysplastic syndromes (MDS), a heterogeneous group of clonal stem cell disorders affecting mostly elderly patients, are characterized by ineffective hematopoiesis, morphological dysplasia of myeloid cells, expansion of bone marrow (BM) myeloid blasts, and a risk of transformation to acute myeloid leukemia (AML). Oxidative stress, aberrant proinflammatory BM microenvironment, activity of myeloid‐derived suppressor cells (MDSCs), increasing intramedullar DNA damage response, and inflammatory cell death all contribute to complex pathogenesis resulting in genetic abnormalities and genome instability of hematopoietic stem and progenitor cells (HSPCs), blast expansion, and enhanced progression of the primary MDS [1, 2]. Chronic inflammatory diseases associated with active innate immune signaling pathways frequently precede MDS (reviewed in ref. [3]). Secondary forms of MDS develop after genotoxic damage of BM due to preceding chemotherapy or radiotherapy [4], suggesting that both groups of MDS share a common genotoxic etiology.

Based on multiple prognostic criteria (e.g., BM blast percentage, platelet and neutrophil counts, and chromosomal abnormalities), MDS patients are assigned to different prognostic groups according to the international prognostic scoring system (IPSS; [5]) or the World Health Organization (WHO) classification [6]. Besides *SF3B1* and *TP53* mutations, the mutational landscape of leukemic blasts remains unutilized in diagnostics and prognosis [7, 8], despite its relevance [8, 9]. The immunological prolifing is currently not applied clinically either [10]. Importantly, biomarkers for the disease monitoring, prediction of therapy‐response, or better stratification of patients are not recognized yet, hence, re‐evaluation of MDS patients' biopsies is currently the only approach to monitor the disease progression [10]. Hypomethylating agents 5‐azacytidine (5‐AC) or 5‐aza‐2′‐deoxycytidine (decitabine) are standard‐of‐care treatments for most of the MDS patients, of which a half responds to the therapy [11].

The IFNγ‐regulated and 5‐AC‐inducible proto‐oncogene *SBSN* [12, 13, 14, 15, 16, 17, 18] plays a prosurviving role in the radio‐ and chemo‐resistant stem cell‐like compartment of some human cancer cells, and its aberrant expression is observed in several human solid malignancies where its presence is linked to tumor progression, aggressiveness, and poor prognosis [19]. Recent findings suggest *SBSN* mediates resistance to lymphocyte‐mediated apoptosis in keratinocytes [20]. Nevertheless, *SBSN* function in physiology and pathology remains unrevealed. The overlap between pathogenetic background of MDS and the known regulatory factors of *SBSN* expression prompted us to investigate the role of *SBSN* in the context of MDS.

In this study, we show aberrant expression of *SBSN* in the BM of MDS patients. The expression of *SBSN* is mediated by myeloid subpopulations, including recently identified crucial mediators of MDS progression, early‐stage MDSCs [21]. The bone marrow SBSN levels anticorrelated with CCL2, a lymphocyte chemokine, and BM T lymphocyte counts. Intriguingly, the highest expression of *SBSN* occurs in the high‐risk disease state. Importantly, secretion of SBSN into BM plasma penetrates into systemic circulation allowing estimation of SBSN in peripheral blood. Overall, these data indicate that *SBSN* expression could contribute to MDS pathology and represents a potential and accessible biomarker of the disease.

## 2 Materials and methods

### 2.1 Chemicals and antibodies

4′,6‐diamidino‐2‐phenylindole (DAPI; Cat. No. D8417); 5‐azacytidine (5‐AC; Cat. No. A2385); 3,3′‐diaminobenzidine (DAB; Cat. No. D8001); doxycycline hydrochloride (Cat. No. D‐9891); DPX Mountant (Cat. No. 06522); Mayer's Hematoxylin Solution (Cat. No. MHS16); hydrogen peroxide 30% (Cat. Co. 31642); phorbol 12‐myristate 13‐acetate (PMA; Cat. No. P8139); poly(ethylene glycol) (PEG; Cat. No. P1458); puromycin (Cat. No. P7255); rabbit serum (Cat. No. R9133); Triton X‐100 (Cat. No. T8787); and TRI Reagent^®^ (Cat. No. 93289) were purchased from Sigma (St. Louis, MO, USA). BamHI (Cat. No. ER0051), EcoRI (Cat. No. ER0271), Lipofectamine RNAiMAX (Cat. No. 13778150), and ProLong™ Gold Antifade (Cat. No. P36930) were purchased from Thermo Fisher Scientific (Waltham, MA, USA). Interferon‐gamma (IFN‐γ; Cat. No. 300‐02) was obtained from PeproTech (Rocky Hill, NJ, USA), SiR‐DNA (Cat. No. SC007) was obtained from Spirochrome (Stein Am Rhein, Switzerland), and Human Suprabasin (SBSN) ELISA kit (Cat. No. MBS9301721) was obtained from MyBiosource (San Diego, CA, USA). Human TruStain FcX™ was purchased from BioLegend (San Diego, CA, USA). The following primary and secondary antibodies were used: anti‐SBSN (Cat. No. HPA067734; dilution 1 : 50; Sigma), anti‐CD11b‐BV421 (Cat. No. 101251; dilution 1 : 200; BioLegend), anti‐HLA‐DR‐APC/Cyanine7 (Cat. No. 307618; BioLegend), anti‐CD33‐PE (Cat. No. 366608; BioLegend), anti‐CD34‐Pacific Blue™ (Cat. No. 343512; BioLegend), anti‐Lineage‐APC (Cat. No. 348803; BioLegend), IgG‐HRP goat anti‐rabbit (Cat. No. 5196‐2504; Bio‐Rad Laboratories, Hercules, CA, USA), Alexa Fluor 568 goat anti‐rabbit (Cat. No. A11036; dilution 1 : 500), and Alexa Fluor 647 goat anti‐rabbit (Cat. No. A‐21244; dilution 1 : 500) were purchased from Invitrogen (Carlsbad, CA, USA).

### 2.2 Cell culture

Human breast carcinoma MCF‐7, human glioblastoma U373, acute myeloid leukemia SKM‐1 and OCI‐M2, and human embryonic kidney 293T (HEK293 cells expressing the large T antigen of SV40; HEK293T) cell lines were obtained from American Type Culture Collection (Manassas, VA, USA). All cell lines were tested for mycoplasma as negative using MycoAlert Mycoplasma Detection Kit (Lonza, Basel, Switzerland) according to manufacturer's instructions. Adherent cells were cultured in Dulbecco's modified Eagle's medium (DMEM; Gibco, Carlsbad, CA, USA) containing 4.5 g·L^−1^ glucose and supplemented with 10% FBS (Gibco), 100 U·mL^−1^ penicillin, and 100 µg·mL^−1^ streptomycin sulfate (Gibco). SKM‐1 cells were cultured in Roswell Park Memorial Institute (RPMI; Gibco) 1640 supplemented with 20% heat‐inactivated FBS (Gibco), 100 U·mL^−1^ penicillin, and 100 µg·mL^−1^ streptomycin (Gibco). OCI‐M2 cells were cultured in Iscove's modified Dulbecco's medium (IMDM; Gibco) supplemented with 20% heat‐inactivated FBS, 100 U·mL^−1^ penicillin, and 100 µg·mL^−1^ streptomycin (Gibco). SKM‐1 or OCI‐M2 cells were seeded at a density of 10 000 cells·cm^−2^ onto 25‐cm^2^ tissue culture flask, treated with 5‐AC (1 µm) or IFN‐γ (5 ng·mL^−1^) for 72 h, and harvested. Cell cultures were kept at 37 °C in 5% CO_2_ atmosphere with 95% humidity.

### 2.3 Patients

In total, three cohorts of patients were analyzed (cohort #1, #2, and #3). Whole BM RNA samples of cohort #1 consisted of MDS *n* = 30, ‘Hematological malignancies’ *n = *19, and ‘Healthy’ *n = *8. Ficoll‐isolated BM mononuclear cell RNA samples of cohort #2 consisted of MDS *n* = 48 and ‘Hematological malignancies’ *n = *11. For patients diagnosis and normalized fold changes of cohorts #1 and #2, see Table S1. BM plasma samples of cohort #3 consisted of MDS *n = *45, MDS 5q‐ *n = *12, AML *n = *10, and non‐MDS *n = *12. For patients diagnosis, see Table S2.

### 2.4 Preparation of the doxycycline‐inducible SBSN‐1 expression vector

MCF‐7 cells were seeded at a density of 22 000 cells·cm^−2^ onto a 60‐mm dish. Twenty‐four hours later, *SBSN* expression was induced with PMA (100 µm for 4 h). After the treatment, total RNA was isolated with an RNeasy Mini Kit (Qiagen Sciences, Germantown, MD, USA) according to the manufacturer's protocol. A complementary DNA (cDNA) strand was synthetized from 1 µg of total RNA with random hexamer primers using a High‐Capacity cDNA Reverse Transcription Kit (Applied Biosystems, Foster City, CA, USA). pLVX Tet‐On *empty* was generated with cloning of a Flag‐tag into a pLVX Tet‐On vector (Clontech, Mountain View, CA, USA) utilizing a set of primers: forward 5′‐GCG AAG AAT TCT TGA CTA CAA AG‐3′, reverse 5′‐CTG GAT CCT TAC TTG TCG TC‐3′ and a subsequent restriction reaction via BamHI and EcoRI (Thermo Fisher Scientific). SBSN isoform 1 (*SBSN‐1*; ENST00000452271.7) was cloned into the Flag‐tag‐containing pLVX Tet‐On *empty* vector utilizing Gibson assembly [22]. Two sets of primers were used as follows: forward *SBSN*: 5′‐TCG TCA TCG TCT TTG TAG TCG GGC ATG ATG TTG GCG ACG C‐3′, reverse *SBSN*: 5′‐TAC CCT CGT AAA ATT CTA GAA TGC ATC TTG CAC GTC TGG T‐3′, forward vector: 5′‐ACC AGA CGT GCA AGA TGC ATT CTA GAA TTT TAC GAG GGT AGG‐3′, reverse vector: 5′‐GCG TCG CCA ACA TCA TGC CCG ACT ACA AAG ACG ATG ACG A‐3′. Generated amplicons underwent a multistep enzymatic reaction of Phusion DNA polymerase (Thermo Fisher Scientific), T5 exonuclease (New England Biolabs, Beverly, MA, USA), and Taq DNA ligase (New England Biolabs). The sequencing control was performed using a set of primers: h*SBSN*: 5′‐CAG GCT GGA AAG GAA GTG GAG A‐3′ and 5′‐CTT GAT GGC TGG AAG ATC CGC T‐3′.

### 2.5 Generation of the stable *SBSN*‐expressing cell line via a lentiviral system

HEK293T cells at 50% confluency were transfected with the solution containing psPAX (7 µg), pMD2.G (4 µg), pLVX Tet‐On *SBSN‐1* or pLVX Tet‐On *empty* (9 µg), and polyethylenimine (83 µg; Polysciences) in Opti‐MEM medium (Gibco). Two days after transfection, the conditioned medium was centrifuged at 3000 ***g*** at 4 °C for 15 min. Subsequently, supernatants containing letiviral particles were precipitated with PEG overnight at 4 °C. After 24 h, precipitated particles were centrifuged at 1500 ***g*** at 4 °C for 30 min. Pellets were resuspended in 1 × PBS and stored at −80 °C until use.

MCF‐7 cells were seeded at a density of 33 000 cells·cm^−2^ onto a 6‐well plate. After 24 h, the cells were transfected with 100 µL of lentiviral particles. The transfected cells were selected with puromycin (2 µg·mL^−1^) for 2 weeks. *SBSN‐1* expression was induced with doxycycline (1 µg·mL^−1^).

### 2.6 RNA interference

U373 cells were seeded at a density of 22 000 cells·cm^−2^ onto a 6‐well plate and transfected concurrently. *SBSN*‐targeting siRNA (Cat. No. s226381; siSBSN; Thermo Fisher Scientific; 5′‐AGA AGG UCA UUG AAG GGA Utt‐3′) or nontargeting siRNA (Cat. No. 4390843; siNC; Thermo Fisher Scientific) was introduced into cells using Lipofectamine RNAiMAX according to the manufacturer's instructions. Forty‐eight hours after the lipofection, the cells were irradiated with a single dose of 2 Gy with the Pantak HF160 (Gulmay, Surrey, UK) X‐ray instrument equipped with Pantak Seifert HF320 generator, MXR‐161 X‐ray tube (Comet AG, Flamatt, Switzerland), and an aluminum filter (using current 1–10 mA). Cells were fixed 24 h postirradiation.

### 2.7 Quantitative real‐time PCR

Total RNA from the bone marrow (BM), mononuclear cells, or *in vitro* culture cell samples, was isolated using TRI Reagent^®^ (Sigma) according to the manufacturer's protocol. cDNA was synthesized from 1 µg of total RNA with random hexamer primers using a High‐Capacity cDNA Reverse Transcription Kit (Applied Biosystems). Quantitative real‐time PCR (RT*–*qPCR) was performed in ABI Prism 7300 (Applied Biosystems) with Syber Select Master Mix (Applied Biosystems). The relative quantity of cDNA was estimated by the ∆∆CT method; data were normalized to GAPDH. Unless stated otherwise, *SBSN* ∆∆CT was normalized to the mean of the ‘Hematological malignancies’ group. Subsequently, fold change (FC) was estimated. Primers used were purchased from Sigma: h*SBSN*: 5′‐CAG GCT GGA AAG GAA GTG GAG A‐3′, 5′‐CTT GAT GGC TGG AAG ATC CGC T‐3′; *GAPDH*: 5′‐GTC GGA GTC AAC GGA TTT GG‐3′, 5′‐AAA AGC AGC CCT GGT GACC‐3′.

### 2.8 Human bone marrow and peripheral blood plasma samples

Human bone marrow or peripheral blood samples were collected into EDTA‐containing tubes. Samples were centrifuged at 1000 ***g*** for 20 min, and then, supernatants were centrifuged at 1000 ***g*** for 5 min. Aliquots of 200 µL of bone marrow or peripheral blood plasma were stored at −80 °C until use. Each aliquot was used only once per analysis.

### 2.9 Isolation of bone marrow mononuclear cells

Mononuclear cells were isolated from patients' bone marrow with Ficoll‐Paque PLUS (GE Healthcare BioSciences AB, Uppsala, Sweden) gradient separation followed by 1 × PBS wash and lysis of the remaining red blood cells by a solution of 144 mmol·L^−1^ NH_4_Cl, 1 mmol·L^−1^ NaHCO_3_, and 1 mmol·L^−1^ EDTA (pH 8.0). For RNA isolation, cells were lysed with TRI Reagent^®^ (Sigma) and processed according to the manufacturer's protocol.

### 2.10 Fluorescence‐activated cell sorting

Ficoll‐isolated BM mononuclear cells were washed with fluorescence‐activated cell sorting (FACS) staining buffer (2% FBS/0.01% sodium azide/1 × PBS) and blocked with Human TruStain FcX™ for 10 min at RT. Subsequently, cells were stained with fluorophore‐conjugated surface antibodies at 4 °C for 20 min, washed twice with FACS staining buffer, and sorted with BD Influx™ cell sorter (BD Bioscience, San Jose, CA, USA). Sorted cell populations were washed with 1 × PBS and lysed with TRI Reagent^®^ (Sigma), then processed according to manufacturer's protocol for RNA and protein isolation.

### 2.11 Enzyme‐linked immunosorbent assay

SBSN levels in patients' bone marrow plasma and peripheral blood were estimated by Human Suprabasin (SBSN) ELISA kit (Cat. No. MBS9301721; MyBiosource). Samples were isolated according to the ELISA kit manufacturer's protocol.

To estimate SBSN in conditioned culture media, MCF‐7 pLVX Tet‐On *SBSN‐1* or *empty* were seeded at a density of 22 000 cells·cm^−2^ onto a 60‐mm dish and cultured with 1 µg·mL^−1^ of doxycycline for 36 h. Conditioned culture media were collected and centrifuged at 1000 ***g*** for 20 min and processed immediately.

### 2.12 Immunohistochemistry of bone marrow sections and smears

Human BM samples were fixed in buffered 4% formaldehyde and decalcified in EDTA pH 7.0. Immunohistochemistry was carried out on 4‐µm paraffin‐embedded bone marrow sections rehydrated via xylene and 100%, 96%, 70%, and 50% ethanol. Antigen retrieval was performed with 10 mm citrate buffer (pH 6.0) in a pressure cooker for 12 min. Endogenous peroxidase activity was blocked with 1% H_2_O_2_ for 15 min. Nonspecific antibody binding was blocked with a normal rabbit serum for 30 min. Sections were incubated with anti‐SBSN antibody (1 : 50) at 4 °C overnight. The next day, slides were washed in 1 × PBS and incubated with the goat anti‐mouse HRP‐conjugated antibody (1 : 500) at RT for 1 h. Sections were then washed in 1 × PBS and incubated with 90 mL of a solution of 50 mm Tris (pH 7.5), 30 mg DAB, and 60 µL 30% H_2_O_2_ in 37 °C for 15 min. Subsequently, sections were washed in 1 × PBS and stained with Mayer's Hematoxylin Solution at RT for 20 min, then washed in water for 7 min, and dehydrated with 50%, 70%, 96%, 100% ethanol, and xylene. Sections were fixed in DPX Mountant. Images were acquired with a Leica DM6000 microscope (Leica Microsystems, Wetzlar, Germany).

Bone marrow smears were fixed in methanol for 15 min. Subsequently, slides were incubated with 0.2% Triton X‐100 for 10 min. Endogenous peroxidase activity, blocking of nonspecific antibody binding, antibody staining, and image acquisition were performed as described in the immunohistochemistry of BM section above.

### 2.13 Indirect immunofluorescence

Bone marrow smears were fixed in methanol for 15 min. Subsequently, slides were incubated with 0.2% Triton X‐100 for 10 min. Nonspecific antibody binding was blocked with a normal rabbit serum for 30 min. BM sections were incubated with anti‐CD11b‐BV421 antibody (1 : 200) and/or anti‐SBSN antibody (1 : 50) at 4 °C overnight. The next day, slides were washed in 1 × PBS and incubated with secondary Alexa Fluor 568‐ or Alexa Fluor 647‐conjugated goat anti‐rabbit antibodies at RT for 1 h. After 1 × PBS and ddH_2_O washes, slides were stained with 1 µg·mL^−1^ DAPI or 1 µm SiR‐DNA, washed with ddH_2_O, and mounted with ProLong™ Gold Antifade Mountant (Thermo Fisher Scientific). Images were acquired with a Leica DM6000 fluorescence microscope or a Leica TCS SP5 confocal laser microscopy system (Leica Microsystems).

siRNA‐treated U373 cells were subjected to indirect immunofluorescence 24 h after irradiation. The cells were fixed with 4% formaldehyde for 15 min, then permeabilized by 0.1% Triton X‐100, washed with 1 × PBS, and blocked with 10% FBS/1 × PBS. Antibody and nuclear DNA staining were performed as described above for BM smears.

### 2.14 Statistical analyses

Data were tested for normality using the Kolmogorov–Smirnov test and are expressed as the mean values ± standard deviation (SD). Pearson's *r* was used for data with normal distribution; Spearman's *r* was used for data with non‐normal distribution. *P‐*values were adjusted using the Benjamini–Hochberg false discovery rate method. One‐way ANOVA or the Kruskal–Wallis test was used for multiple comparisons followed by multiplicity adjusted Tukey's *post hoc* or the Dunn's test. The two‐tailed Mann–Whitney *U*‐test, the two‐tailed unpaired *t*‐test, and the two‐tailed paired *t*‐test were used accordingly for two‐sample comparisons. The asterisks represent *P* < 0.05 (*), *P* < 0.01 (**), *P* < 0.001 (***). All analyses were performed with the graphpad prism software (San Diego, CA, USA) and the r software package [23].

### 2.15 Study approval

Informed written consent has been obtained from all patients in compliance with the Declaration of Helsinki and approved by the Ethics Committee of the General Hospital Prague.

## 3 Results

### 3.1 *SBSN* mRNA is elevated in bone marrow of MDS patients

**Fig. 1:**
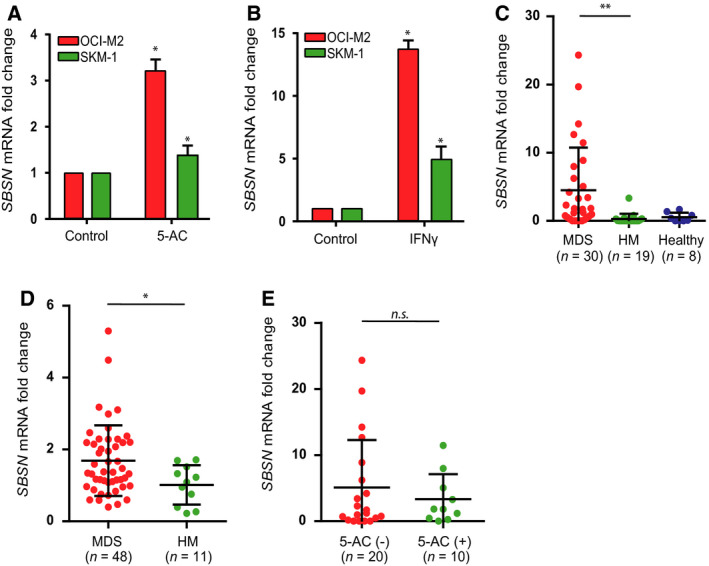
SBSN transcript is induced *in vitro* by IFN‐γ and 5‐AC treatment and is present in MDS patients' bone marrow. RT–qPCR quantification of *SBSN* mRNA fold change in OCI‐M2 and SKM‐1 leukemic cell lines treated with (A) 5‐AC (1 µm) and (B) IFN‐γ (5 ng·mL^−1^) for 72 h, with *n* = 3 per group. RT–qPCR quantification of *SBSN* mRNA fold change in (C) whole bone marrow samples of cohort #1 three groups of donors ‘MDS’ (*n* = 30), ‘HM’ (*n* = 19), and ‘Healthy’ (*n* = 8) and (D) in cohort #2 bone marrow mononuclear cell samples of two groups of donors ‘MDS’ (*n* = 48) and ‘HM’ (*n* = 11). (E) RT–qPCR quantification of *SBSN* mRNA fold change in whole bone marrow samples of newly diagnosed [‘5‐AC(−)’, *n* = 20] and therapy‐undergoing [‘5‐AC(+)’, *n* = 10] patients. The two‐tailed Mann–Whitney *U‐*test was used for two‐sample comparison. For patients' samples, one‐way ANOVA was used for multigroup comparisons followed by the Tukey's *post hoc* test. The two‐tailed unpaired *t*‐test was used for two‐group comparisons. Results are expressed as the means ± SD; *P < 0.05, **P < 0.01.

Given the *SBSN* expression can be induced in some solid tumor cells with 5‐aza‐2′‐deoxycytidine (decitabine) and 5‐azacytidine (5‐AC; Vidaza^®^), both used for therapy of high‐risk MDS patients [13, 24], we first analyzed whether 5‐AC can induce *SBSN* expression in OCI‐M2 and SKM‐1 MDS/AML‐derived cell lines *in vitro*. Indeed, treatment with 5‐AC (1 µm, 72 h) resulted in significant elevation of *SBSN* mRNA in both OCI‐M2 and SKM‐1 cells (Fig. 1A). Interferon‐gamma (IFN‐γ), another recently identified inducer of *SBSN* expression [13], also elevated *SBSN* mRNA levels in both cell lines (Fig. 1B), thus expanding the previous findings of the *SBSN* upregulation also to cells of hematopoietic origin.

This result prompted us to search for the presence of *SBSN* transcripts in the BM cells of three groups of donors: (a) patients with MDS, (b) patients with hematological malignancies (HM; lymphomas, leukemias, and MDS‐transformed AML), and (c) healthy BM donors (cohort #1; see Table S1 for patients' diagnosis). Intriguingly, *SBSN* mRNA level in the MDS BM patients' samples (*n* = 30) was significantly higher compared to either the HM group (*n* = 19; *P* = 0.0053; Fig. 1C) or healthy donors (*n* = 8) in which the level of *SBSN* mRNA was low or undetectable. When analyzing BM mononuclear cells (BM‐MNC; cohort #2; see Table S1 for patients' diagnosis), we detected significantly higher (*P* = 0.033) *SBSN* mRNA levels in MDS (*n* = 48) compared to HM patient group (*n* = 11; Fig. 1D).

As 5‐AC induced *SBSN* expression *in vitro*, we investigated next the effect of 5‐AC therapy on *SBSN* expression by comparison of the two groups of patients from cohort #1, newly diagnosed MDS patients without hypomethylating therapy and MDS patients undergoing 5‐AC therapy at the time of BM sample collection. Our dataset did not show significant difference in *SBSN* mRNA fold change between newly diagnosed patients and 5‐AC‐treated MDS groups (*P* = 0.47; Fig. 1E), suggesting aberrant expression of *SBSN* is driven by alterations of intrinsic factors in MDS BM and, possibly, by the disease progression. Nevertheless, due to a limited sample size it still cannot be concluded whether the 5‐AC therapy contributes to further elevation of SBSN expression in the patients undergoing such therapy.

Collectively, these data show that the transcription of *SBSN* can be induced in human leukemic cell lines by IFN‐γ or 5‐AC. Importantly, *SBSN* mRNA levels are increased in the bone marrow of MDS patients.

### 3.2 SBSN protein is increased in bone marrow and peripheral blood plasma of MDS patients

**Fig. 2:**
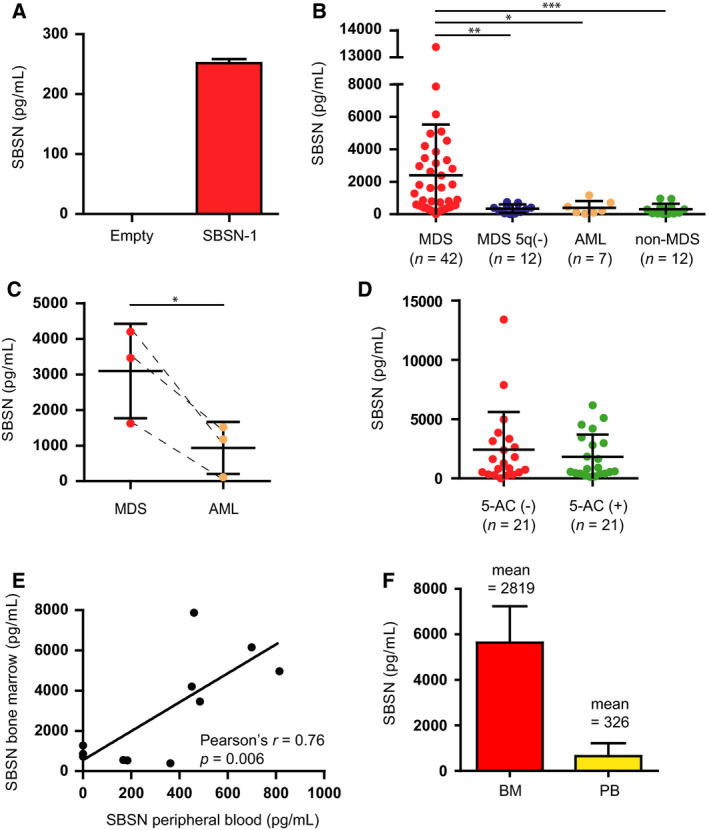
SBSN is a secreted protein present in MDS patients' bone marrow and peripheral blood. Anti‐SBSN ELISA detection of SBSN protein levels (pg·mL^−1^) (A) in the conditioned medium of MCF‐7 pLVX Tet‐On *empty*/*SBSN‐1* cell lines and (B) in bone marrow plasma of cohort #3 four groups of patients ‘MDS’ (*n* = 42), ‘MDS 5q(−)’ (*n* = 12), ‘AML’ (*n* = 7), and ‘non‐MDS’ (*n* = 12). Comparison of SBSN protein levels in BM of MDS patients prior (‘MDS’) and after (‘AML’) leukemic transformation (C). BM SBSN protein levels in MDS patients without 5‐AC therapy [‘5‐AC(−)’; *n* = 21] and patients undergoing 5‐AC therapy [‘5‐AC(+)’; *n* = 21] (D). Correlation between *SBSN* protein levels (pg·mL^−1^) (E) in bone marrow plasma and peripheral blood of MDS patients (*n* = 11) and (F) the mean comparison. The Kruskal–Wallis test was used for multiple comparisons followed by Dunn's multiple comparison test. The two‐tailed paired *t*‐test was used for statistics in panel C. The two‐tailed Mann–Whitney *U‐*test was used for two‐sample comparison. Pearson's correlation coefficient was used for correlation statistics. Results are expressed as the means ± SD; *P < 0.05, **P < 0.01.

As we detected aberrantly elevated *SBSN* mRNA in the MDS BM, and since SBSN is a secreted protein [14, 15, 16, 17, 18], we next examined SBSN in BM plasma. First, we evaluated the specificity of the anti‐SBSN ELISA kit using doxycycline‐inducible expression of *SBSN‐1* isoform in MCF‐7 cells. Indeed, SBSN was detected specifically in the culture medium conditioned by doxycycline‐induced *SBSN‐1* expressing cells (Fig. 2A). Next, we measured SBSN protein levels in BM plasma of MDS, MDS 5q‐syndrome, AML, and non‐MDS (thrombocytopenia, multiple myeloma, and healthy donors) patients (cohort #3; for patients' diagnosis, see Table S2). Intriguingly, SBSN levels in BM samples of MDS (*n = *42) were significantly higher compared to the MDS 5q‐ (*n* = 12; *P* < 0.001), MDS‐transformed AML (*n* = 7; *P* = 0.0106), and non‐MDS (*n* = 12; *P* = 0.0004) groups (Fig. 2B). Lower SBSN levels in BM plasma of AML patients in comparison with MDS patients prompted us to analyze SBSN protein levels of MDS patients prior to the leukemic transformation and in the AML state. Indeed, analysis of a group of three patients before and after the AML transformation showed SBSN protein levels significantly diminished after the AML transformation (Fig. 2C). Similarly to *SBSN* transcript levels, no significant difference in BM SBSN protein levels of MDS patients undergoing 5‐AC therapy during the sample acquisition [5‐AC(+)] and without the treatment [5‐AC(−)] was observed (*P* = 0.46; Fig. 2D).

Estimation of SBSN protein in PB plasma showed a significant correlation of SBSN levels paired in BM and PB plasma (*n* = 11; Pearson's *r* = 0.76; *P* = 0.006; Fig. 2E). Note, the SBSN levels in BM plasma were on average 8 times higher compared to PB plasma (Fig. 2F), suggesting BM as a possible source of SBSN in MDS.

Collectively, these data show high expression and secretion of SBSN protein in BM and PB plasma of MDS patients.

### 3.3 MDS patients with high SBSN protein levels correspond to poor prognosis groups

**Fig. 3:**
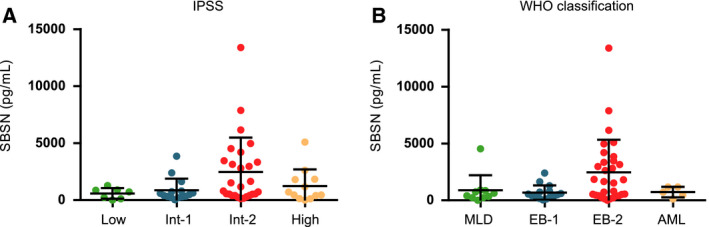
High SBSN levels correspond to MDS poor prognosis groups. SBSN protein levels (pg·mL^−1^) assigned to patients' prognostic groups according to (A) the international prognostic scoring system of MDS (IPSS) and (B) The World Health Organization (WHO) classification of MDS, with *n* = 7 for Low IPSS, *n* = 15 for Int I IPSS, *n* = 27 for Int II, *n* = 12 for High IPSS, and with *n* = 10 for MLD, *n* = 15 for EB‐1, *n* = 31 for EB‐2, *n* = 5 for AML.

To estimate whether there is a link between *SBSN* expression and patients' disease state and prognosis, we assigned *SBSN* protein levels of patients' cohort #3 to prognostic groups of the international prognostic scoring system of MDS (IPSS [5, 25]) or WHO MDS classification [6, 26]. Notably, the patients with the highest BM plasma SBSN levels belonged to the IPSS Int‐2 group (Fig. 3A) and into the poor prognosis WHO EB‐2 group (Fig. 3B).

Taken together, MDS patients with high BM SBSN protein levels correspond to poor prognosis groups of both IPSS and WHO classifications of MDS patients.

### 3.4 *SBSN* is expressed in subpopulations of polymorphonuclear and mononuclear cells in the bone marrow of MDS patients

**Fig. 4:**
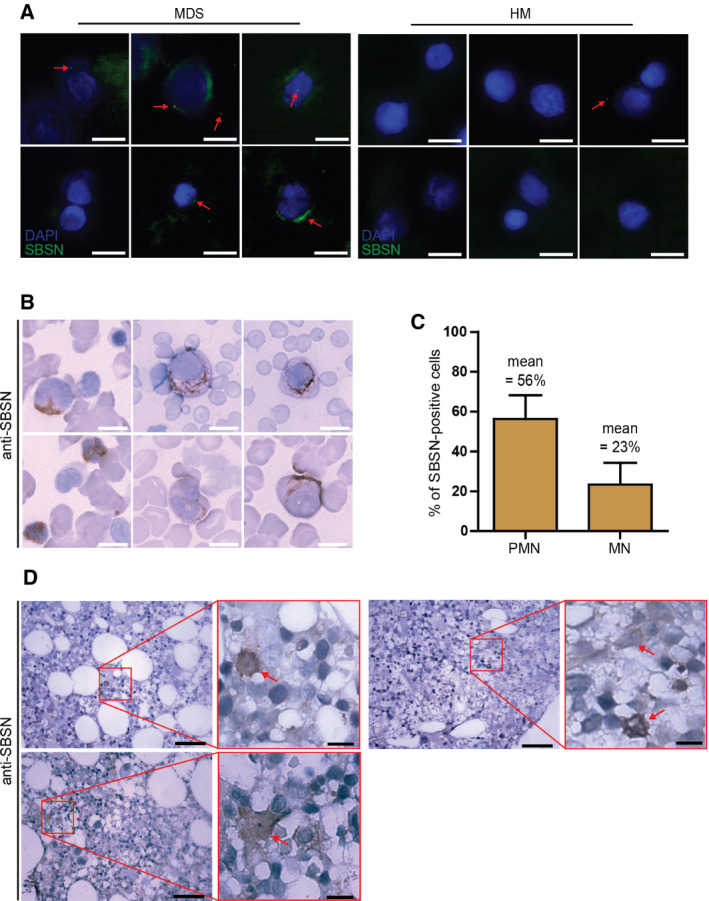
Polymorphonuclear and mononuclear cells express *SBSN* in bone marrow of MDS patients. (A) Immunofluorescence detection of SBSN (green) using anti‐SBSN and Alexa Fluor 647 goat anti‐rabbit in bone marrow smears of the MDS (EB‐2; *n* = 3) and HM (Hodgkin's lymphoma; *n* = 3) groups. Nuclei were stained with DAPI (blue). Scale bar, 10 µm. (B) Immunohistochemistry of SBSN in bone marrow smears of MDS patients using anti‐SBSN antibody and IgG‐HRP goat anti‐rabbit (EB‐1, and EB‐2; *n* = 3; scale bar, 10 µm) and (C) quantification of SBSN‐positive polymorphonuclear (PMN; 56%) and mononuclear (MN; 23%) cells in MDS patients' bone marrow smears. 100 PMN and 100 MN cells were counted from each sample (*n* = 3). Results are expressed as the means ± SD. (D) Immunohistochemistry of SBSN using anti‐SBSN antibody and IgG‐HRP goat anti‐rabbit in bone marrow sections of the MDS group (MDS‐MLD and 5q‐; *n* = 3). Scale bars, 50 µm (larger image) and 10 µm (smaller image).

To identify a specific cell type responsible for the production and secretion of SBSN into BM of MDS patients, we first analyzed the presence of SBSN in BM smears by indirect immunofluorescence (IF). Specificity of SBSN antibody for IF was verified using RNA interference in the U373 glioblastoma cell line expressing *SBSN* after exposure to ionizing radiation (see green cytoplasmic signal for SBSN; Fig. S1A). As shown in Fig. 4A, cytoplasmic signal [13] of SBSN can be observed in subpopulation of cells in MDS BM smears. In contrast, the SBSN signal was only infrequently present in HM samples. Morphologically, SBSN‐positive cells resembled mononuclear (MN) or immature polymorphonuclear (PMN) cells (Fig. 4B). However, SBSN‐negative subsets of both MN and PMN cells were also present in the same patient samples (Fig. S1B), indicating *SBSN* expression is limited to a subpopulation of MN and PMN cells. As quantified in Fig. 4C, 56% of PMN cells and 23% of MN cells were SBSN‐positive. Similarly, SBSN‐positive cells were detected in BM section of MDS patients (Fig. 4D).

Altogether, these results show that SBSN is expressed in BM of MDS patients in subpopulations of polymorphonuclear and mononuclear cells.

### 3.5 *SBSN* is expressed in myeloid cell compartment of MDS BM

**Fig. 5:**
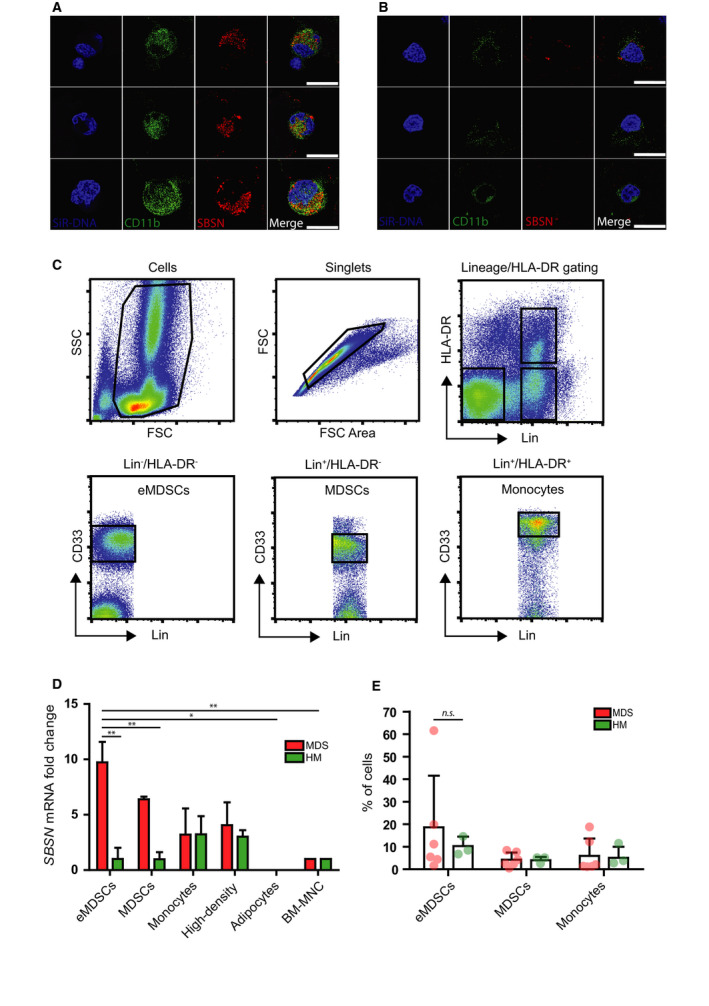
Myeloid cells express *SBSN* in bone marrow of MDS patients. Immunofluorescence detection of CD11b (green) using anti‐CD11b‐BV421, SBSN (red) using anti‐SBSN and Alexa Fluor 568 goat anti‐rabbit in bone marrow smears of (A) MDS (EB‐2) and (B) MDS 5q‐syndrome patients. Nuclear DNA is stained by SiR‐DNA (blue), with *n* = 3 per group. Scale bar, 10 µm. (C) Gating strategy used for isolation of eMDSCs, MDSCs, and monocyte populations from the bone marrow of MDS (*n* = 6) and ‘hematological malignancy’ patients (HM; multiple myelomas, T‐cell lymphoproliferation, chronic anemia; *n* = 4). (D) *SBSN* mRNA levels of BM myeloid subpopulations eMDSCs [Lin^−^ (CD3, 14, 16, 19, 20, 56) HLA‐DR^−^, CD33^+^], PMN‐/MN‐MDSCs (MDSCs; Lin^+^, HLA‐DR^−^, CD33^+^), monocytes (Lin^+^, HLA‐DR^+^, CD33^++^), adipocytes, and granulocyte‐containing high‐density fraction in MDS (*n* = 4) and HM (multiple myelomas and T‐cell lymphoproliferation; *n* = 3) estimated after cell sorting by RT–qPCR. (E) Comparison of the bone marrow subpopulation percentage in MDS (*n* = 6) and HM (multiple myelomas and chronic anemia; *n* = 3) patients. For patients' samples, one‐way ANOVA was used for multigroup comparisons followed by the Tukey's *post hoc* test. Results are the means ± SD; *P < 0.05, **P < 0.01.

Since PMN phenotype is characteristic for the myeloid lineage‐derived cells, we performed immunofluorescence staining of BM smears with anti‐CD11b, the marker of myeloid lineage, and anti‐SBSN. Indeed, SBSN‐positive cells were also positive for CD11b (Fig. 5A), implicating that the cells of the myeloid origin are the source of *SBSN* expression in BM of MDS patients. Consistently with relatively low SBSN levels in BM plasma of MDS 5q‐, BM smears of 5q‐syndrome patients showed very low SBSN staining (Fig. 5B).

To further verify the expression of *SBSN* in BM myeloid cells, next we sorted the myeloid subpopulations from Ficoll‐isolated BM‐MNC by FACS (for gating strategy, see Fig. 5C) of MDS and HM (multiple myelomas and T‐cell lymphoproliferation) patients and compared *SBSN* mRNA levels to the source BM‐MNC to determine the level of enrichment (Fig. 5D). We also determined *SBSN* mRNA levels in Ficoll‐isolated high‐density granulocyte‐containing cellular fraction (‘High‐density’) and BM adipocyte tissue (‘Adipocytes’). *SBSN* mRNA was significantly enriched in early‐stage myeloid‐derived suppressor cells [eMDSCs; Lin^−^ (CD3, 14, 16, 19, 20, 56) HLA‐DR^−^, CD33^+^] of MDS patients. We also detected *SBSN* mRNA in mature PMN‐/MN‐MDSC‐containing subpopulation (MDSCs; Lin^+^, HLA‐DR^−^, CD33^+^). Monocyte subpopulation (Lin^+^, HLA‐DR^+^, CD33^++^) and high‐density cells showed similar but lower levels of *SBSN* mRNA in both MDS and HM patients as compared to eMDSCs. Note, the number of MDSCs in MDS did not significantly differ from HM group (Fig. 5E) indicating the higher presence of SBSN in BM of MDS patients is due to its enhanced expression in subpopulations of MDSCs.

Collectively, aberrant expression of *SBSN* is mediated by the myeloid cell compartment in BM of MDS patients, predominantly by early‐stage MDSCs.

### 3.6 *SBSN* expression in the bone marrow of MDS correlates negatively with chemokine CCL2 levels and lymphocyte count and positively with blast count

**Fig. 6:**
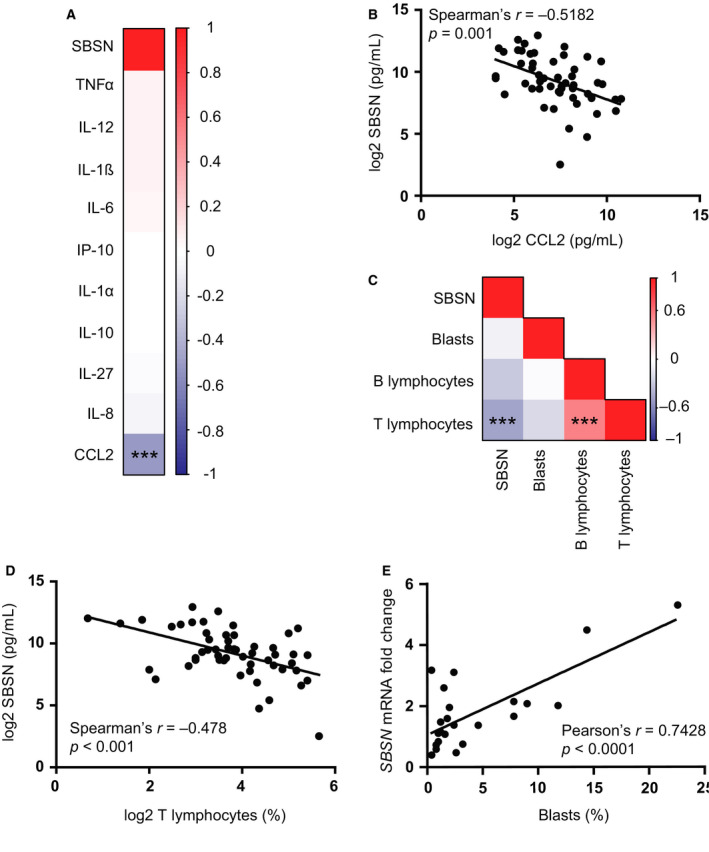
SBSN protein levels anticorrelate with MDS patients' bone marrow CCL2 chemokine levels and T lymphocyte count. (A) Correlation matrix of log2 SBSN protein levels (pg·mL^−1^) and TNF‐α, IL‐12, IL‐1β, IL‐6, IP‐10, IL‐1α, IL‐10, IL‐27, IL‐8, and CCL2 cytokine log2 levels (pg·mL^−1^), ***P < 0.001. (B) Correlation between log2 SBSN protein (pg·mL^−1^) and log2 CCL2 levels (pg·mL^−1^) in bone marrow of MDS, MDS 5q‐, and AML patients (*n* = 52). Spearman's correlation coefficient was used for correlation statistics. (C) Correlation matrix of log2 SBSN protein levels (pg·mL^−1^) and T, B lymphocytes and blast percentages in BM, ***P < 0.001. (D) Correlation between log2 BM SBSN protein levels (pg·mL^−1^) and log2 BM T lymphocyte percentage of MDS, MDS 5q‐, and AML patients (*n* = 53). (E) Correlation between SBSN mRNA fold changes of MDS patients' mononuclear cell samples and corresponding blast count in percentage. Spearman's correlation coefficient and Pearson's correlation coefficient were used for correlation statistics of non‐normal data distribution (D) and normal data distribution (E), respectively. *P*‐values were adjusted using the Benjamini–Hochberg false discovery rate method.

As in cancer cells *SBSN* expression can be controlled by cytokine signaling [13], and levels of several cytokines are altered in BM of MDS (for a review, see ref. [2]), we further explored whether there is a relationship of *SBSN* expression with cytokine levels. Here, the SBSN protein levels were estimated in cohort #3 of MDS, MDS 5q‐, and MDS‐transformed AML patients previously analyzed by us for the level of inflammatory cytokines [27]. Correlation analysis of ten cytokines (IL‐1α, IL‐1β, IL‐6, IL‐8, IL‐10, IL‐12, IL‐27, IP‐10, CCL2, and TNF‐α) and SBSN protein levels (*n* = 52; Fig. 6A) showed significant negative correlation with proinflammatory chemokine CCL2 (Spearman's *r* = −0.5182; *P* < 0.001; Fig. 6B), a chemokine modulating the function of monocytes, basophils, and lymphocytes [28].

This prompted us to investigate the relation of BM plasma SBSN levels with T and B lymphocytes, and blast counts in BM of patients' cohort #3 (Fig. 6C). Interestingly, BM SBSN protein levels showed significant negative correlation with BM T lymphocytes (*n* = 53; Spearman's *r* = −0.478; *P* < 0.001; Fig. 6D). Anticorrelation was achieved when we compared BM SBSN protein levels and BM B lymphocyte levels, but its significancy was lost after the *P‐*values adjustment (Fig. S1C). However, BM T lymphocytes showed significant correlation with BM B lymphocytes (Fig. S1D). In line with its role as a chemokine, CCL2 showed significant correlation with the number of BM T cells (Fig. S1E), but not with B cells (Fig. S1F). BM SBSN protein levels did not show correlation with BM blasts, but SBSN mRNA of patients' cohort #1 showed positive correlation with blast percentage (*n* = 23; Pearson's *r* = 0.7428; *P* < 0.0001; Fig. 6E).

Altogether, *SBSN* expression in BM of MDS and AML patients correlates positively with BM blast count and negatively with CCL2 levels and T lymphocyte count.

## 4 Discussion

Chronic inflammatory stimuli, innate immune signaling, and genetic instability of mutant HSPCs drive traits of myelodysplastic syndromes, such as multilineage cytopenia, inflammatory cell death, and evolution of mutant clones as well as transformation into acute myeloid leukemia. Somatic mutations and chromosomal anomalies of HSPCs responsible for aberrant expression programs and genome instability are well documented [29, 30, 31]. Inflammatory signaling, inefficiency of the immune system, and pyroptotic cell death are now thought to represent crucial factors for MDS progression [2, 29]. Proinflammatory signals and innate immune response promote positive feedback through expansion of MDSCs, enhancing cytokine signaling and subsequent genotoxic conditions essential for blast accumulation and mutagenesis [29]. The later stages of MDS progression are accompanied with strong immunosuppression mediated by MDSCs, intensifying nonpermisive BM conditions and immune evasion of blasts, thus promoting leukemic transformation [10, 21, 29, 32].

In a recent work, Winter *et al*. summarized the current needs for improved clinical treatments of MDS. An urgent call for identification of biomarkers and better stratification of MDS patients is apparent. Since MDS is a heterogeneous group of diseases, the universal biomarker is not likely to be identified, thus revealing individuals' or subgroup biomarkers is of great interest. Neither the immune profile, nor BM cytokine alterations, the essential features of MDS progression and prognosis, are addressed in the current prognostic systems. So far, immune checkpoint inhibitor (ICI) monotherapies in MDS did not provide appreciated results, and hence, biomarkers predicting efficiency of ICI therapies might improve patients' stratification in MDS clinical trials [10].

Here, we show the elevated expression of a recently identified proto‐oncogene *SBSN* in BM of MDS patients. Although the molecular function of *SBSN* remains elusive, a growing number of studies report increased *SBSN* expression in human malignancies in association with therapy resistance and cancer cell survival [14, 19, 33, 34]. The cause of aberrant *SBSN* expression in BM of MDS donors is unclear, and hence, its participation in pathogenesis of MDS is difficult to unravel at present. *SBSN* is shown to promote‐catenin signaling in human esophagus cancer via unknown mechanisms and its expression is a marker of colon cancer tumor endothelial cells, implicating that *SBSN* aberrant expression in both tumor cells and tumor‐affected healthy cells is associated with cancer malignancy. Demethylation of the *SBSN* gene promoter is associated with metastatic diseases [24], and 5‐AC, a demethylating agent used for MDS treatment, promotes *SBSN* expression in lung cancer cells via induction of the transcription regulator BORIS [34]. As we found that both IFN‐γ and 5‐AC induced transcription of *SBSN* in leukemic cells lines OCI‐M2 and SKM‐1, changed cytokine environment [35, 36] and epigenetic state [37] in BM of MDS patients could be putative drivers of *SBSN* expression. However, we did not find positive correlation with inflammatory cytokine levels in BM or 5‐AC therapy. Further work is needed to evaluate whether there is functional link between, for example, IFN‐γ, known to be increased in BM of MDS patients [35], and *SBSN* expression. The fact that the presence of SBSN is not a consequence of the 5‐AC treatment implies that the major factors behind the observed aberrant expression are endogenous, reflecting the disease state, thereby further supporting the potential value of SBSN as a genuine biomarker of MDS. Higher SBSN levels in MDS IPSS int‐2 and EB‐2 groups indicate SBSN level elevation is a feature of the high‐risk MDS subgroup. Intriguingly, the decrease in preceding higher SBSN levels in the course of leukemic transformation indicates a utilization potential of monitoring disease progression via SBSN levels.

In attempt to unravel the cell source of SBSN in BM of MDS, we identified CD11b^+^ myeloid polymorphonuclear (PMN) and mononuclear (MN) cells to express *SBSN*. Further analysis revealed the highest *SBSN* mRNA levels in early‐stage MDSCs (eMDSCs) compared to other MDS BM cell subpopulations including MDSCs, monocytes, granulocytes, and adipocytes. Monocytes and granulocytes showed similar SBSN levels in MDS in comparison with other hematological malignancies (HM) such as multiple myeloma and T‐cell lymphoproliferation, indicating both cell types are responsible for basal SBSN levels in BM rather than for MDS‐specific expression generated by eMDSCs and MDSCs. Importantly, we did not find significant difference in abundance of eMDSCs and MDSCs among MDS and HM groups. This indicates that enhanced expression of *SBSN* rather than elevated numbers of MDSCs is responsible for higher SBSN levels in BM of MDS.

Generally, long‐lasting inflammatory signaling known to be mediated by tumor cell growth and tumor microenvironment, including the MDS bone marrow niche, promotes accumulation of immunosuppressive MDSCs, and thus facilitates tumor cell survival and boosts chronic inflammation [38]. MDSCs suppress the function and migration potential of T lymphocytes via multiple mechanisms, including arginine depletion, production of reactive oxygen species, and nitration of chemokines [38, 39]. Furthermore, to mediate immunosuppressive activity mouse monocytic MDSCs require the active IFN‐γ‐STAT1‐IRF1 pathway [40], the pathway likely responsible for *SBSN* expression in human cancer cell lines [13]. Indeed, while IFN‐γ showed altered levels in BM of MDS [41, 42], additional work is needed to elucidate the possible role of IFN‐γ signaling in *SBSN* expression in this disease context. MDSCs in MDS derive from healthy HSPCs lacking mutations present in malignant blasts [21], indicating that occurrence and activity of MDSCs is mediated via inflammatory milieu, a hallmark of MDS BM. It should be stressed, however, that the detection of MDSCs in human samples is relatively difficult, and the presence of MDSCs remains largely unexplored in MDS patients' BM. The myeloid origin of MDSCs grants them with the polymorphonuclear (PMN‐MDSCs; CD11b^+^, CD14^−^, CD15^+^, CD33^+^, HLA‐DR^−^) or mononuclear (MN‐MDSCs; CD11b^+^, CD14^+^, CD15^−^, CD33^+^, HLA‐DR^−^) phenotype. Additionally, eMDSCs (Lin^−^, CD33^+^, HLA‐DR^−^), putative precursors of PMN‐/MN‐MDSCs, with a detrimental role in MDS, are also defined [21]. The origin of the MDSCs is still a subject of debate. MDSCs were originally described as myeloid precursor‐derived cells with a potential to re‐enter differentiation program and become mature dendritic cells/macrophages or granulocytes [43]. Later findings show that MDSCs represent immunosuppressive phenotype of monocytes and granulocytes, respectively [44]. Both observations describe the diverse nature of myeloid cells in response to infection, wound healing, or cancer.

Myeloid‐derived suppressor cells were shown to promote multiple lineage cytopenia, a crucial aspect of the progressing MDS [21]. The frequencies of MDSCs in BM of MDS or AML patients vary greatly [21, 45] with elevated MDSCs observed in int‐/high‐risk MDS patients compared to low‐risk patients [32]. In low‐risk MDS, eMDSCs promote inflammatory cell death, while strong immunosuppressive environment is established by relatively abundant eMDSCs in later stages of the disease. Herein observed subtype of MDSCs specifically expressing *SBSN* showed significant anticorrelation with proinflammatory CCL2, the lymphocyte chemokine, and BM T lymphocyte count, which indicate BM immunosuppressive milieu in a group of high‐risk MDS. Therefore, SBSN can possess ICI therapy‐predictive potential that needs to be further evaluated.

Importantly, SBSN in peripheral blood follows SBSN levels in BM, thus SBSN in PB could provide useful information about its BM status. Additionally, the correlation of *SBSN* transcript levels with percentage of MDS patients' blasts and significantly higher *SBSN* protein levels in the MDS in general suggest that SBSN occurrence in the MDS patients might contribute directly to MDS pathogenesis and the disease progression.

Progression of MDS is linked to abrogation of T‐cell program and impairment of CD4^+^ cell development [46]. Indeed, lymphopenia has been recently shown to be a negative prognostic marker, especially in low‐risk MDS [47]. Moreover, CCL2 is associated with Th2 polarization and promotes Th2‐mediated *IL‐4* expression [28, 48]. Notably, downregulation of *SBSN* sensitizes keratinocytes to Th2‐mediated apoptosis triggered by IL‐4 and IL‐13 [20]. The protective role of SBSN in keratinocytes is in agreement with its recently proposed cell death‐suppressing ability during radio‐ and chemo‐therapy of cancer cells [13]. IL‐4 induces cell death in AML patients' blasts without promoting the effect in healthy HSPCs [49], and IL‐4 levels are associated with longer survival of MDS patients [35]. Whether SBSN inhibits IL‐4‐mediated cell death in leukemic blasts and thus provides resistance of malignant clones to the immune system in MDS requires further investigation.

It can be proposed *SBSN* expression is likely a myeloid cell response to yet unknown stimuli. A recent study [50] reports an interaction of SBSN in MDS PB plasma with LRG1 receptor. This strengthens the importance and clinical relevance of our observation and proposes a mechanism of SBSN action. Notably, granulocyte‐secreted LRG1 suppresses TGFβ‐mediated inhibition of CD34^+^ and myeloid progenitor cell proliferation [51]; therefore, *SBSN* could promote the expansion of myeloid and blast cells. Besides β‐catenin signaling, SBSN induces Akt and p38 activation *in vitro* [33, 52] via a so far unknown mechanism, and hence, *SBSN* potential to promote prosurvival signaling in BM of MDS should be explored in future studies.

## 5 Conclusions

In conclusion, the expression of *SBSN* by myeloid cells in the bone marrow of MDS patients is associated with negative prognostic traits and its presence in peripheral blood makes it a perspective candidate for accessible biomarker in a subgroup of high‐risk MDS patients.

## Conflict of interest

The authors declare no conflict of interest.

## Author contributions

MP involved in design, realization, and analysis of experiments, and wrote the manuscript. SH and AM involved in design, realization, and analysis of experiments. MV, HP, RB, OF, JS, TS, and AJ collected patient samples and/or prepared the samples. BS analyzes the data. JB and ZH designed the study, analyzed the data, and wrote the manuscript. All authors discussed, interpreted the results, and approved the manuscript.

## Supporting information


**Fig. S1.** Detection of SBSN in irradiated glioblastoma cells, representation of SBSN‐negative cells in MDS BM, and correlations of B cells, T cells, and CCL2 with SBSN levels.

**Table S1.** Clinical characteristics of ‘MDS’ and ‘hematological malignancies’ patients groups of Cohort #1 and #2 including *SBSN* mRNA fold change.

**Table S2.** Clinical characteristics of ‘MDS’ and ‘hematological malignancies’ patients groups of Cohort #3 including bone marrow blasts and T cells percentages, with bone marrow and peripheral blood SBSN concentrations.
