# Supplementary figures and images for: Aberrantly elevated suprabasin in the bone marrow as a candidate biomarker of advanced disease state in myelodysplastic syndromes
Source: Mol Oncol. 2020 Aug 11;14(10):2403–19. doi: 10.1002/1878-0261.12768 (PMC7530796; doi:10.1002/1878-0261.12768)

# Supplementary Figure 1

A

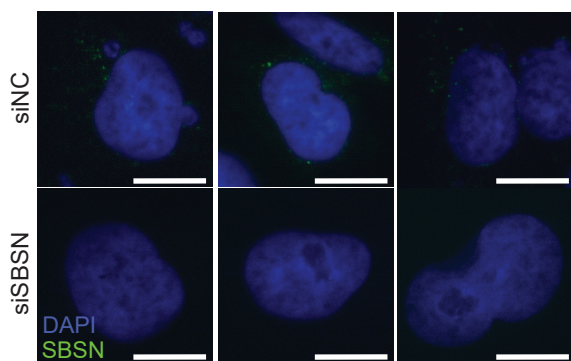

B

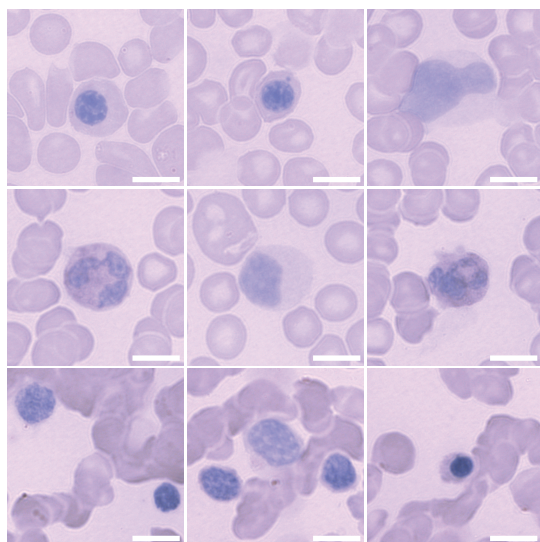

C

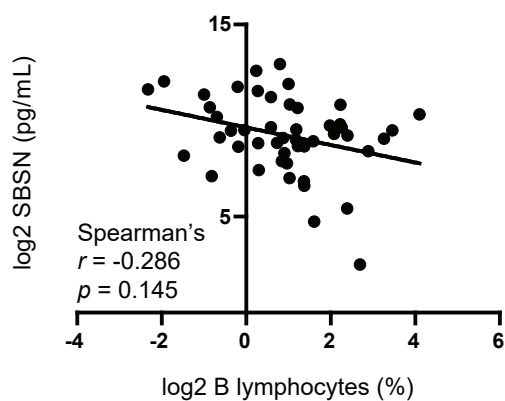

D

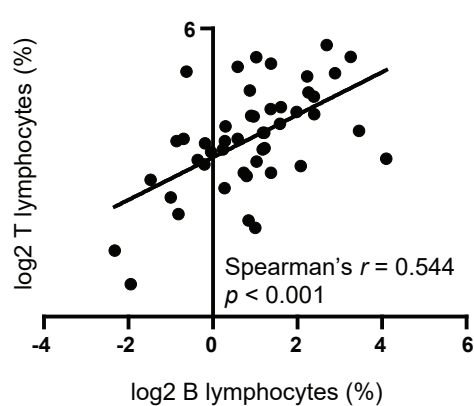

E

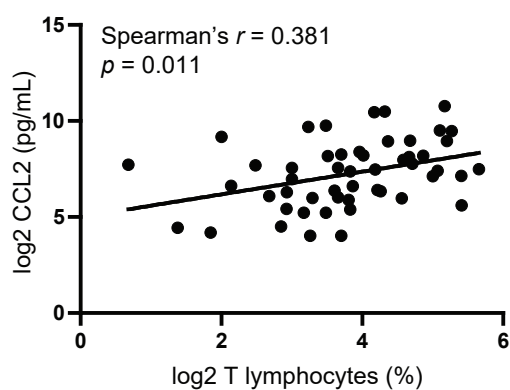

F

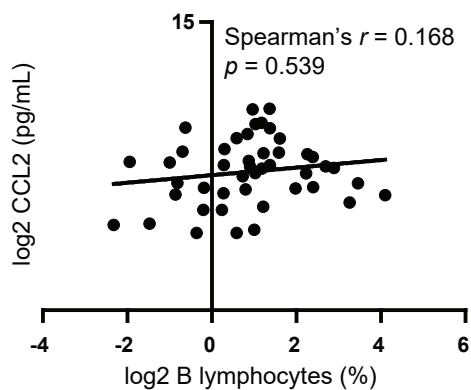

Supplement: Supplementary file 1 — Fig. S1. Detection of SBSN in irradiated glioblastoma cells, representation of SBSN‐negative cells in MDS BM, and correlations of B cells, T cells, and CCL2 with SBSN levels. [file MOL2-14-2403-s001.pdf]
