# Supplementary material for: Aberrantly elevated suprabasin in the bone marrow as a candidate biomarker of advanced disease state in myelodysplastic syndromes
Source: Mol Oncol. 2020 Aug 11;14(10):2403–19. doi: 10.1002/1878-0261.12768 (PMC7530796; doi:10.1002/1878-0261.12768)
Supplement: Supplementary file 2 — Table S1. Clinical characteristics of ‘MDS’ and ‘hematological malignancies’ patients groups of Cohort #1 and #2 including SBSN mRNA fold change. [file MOL2-14-2403-s002.docx]

| Clinical characteristics of 'MDS' and 'hematological malignancies' patients groups of Cohort #1 and #2 including SBSN mRNA fold change. | | | |
| --- | --- | --- | --- |
| Cohort #1 | | | |
| MDS group (*n* = 30) | | | |
| Disease State | Diagnosis | *SBSN* mRNA Fold change | Treatment |
| MDS | MDS-SLD | 1.17 | 5-AC (Vidaza) |
| MDS | MDS-EB-2 | 0.45 | 5-AC (Vidaza) |
| MDS | MDS-EB-2 | 3.27 | 5-AC (Vidaza) |
| MDS | MDS-EB-2 | 0.97 |  |
| MDS | MDS-EB-2 | 1.81 | 5-AC (Vidaza) |
| MDS | MDS-EB-2 | 0.75 |  |
| MDS | MDS-EB-1 | 0.26 | 5-AC (Vidaza) |
| MDS | MDS-EB-2 | 8.87 |  |
| MDS | MDS-MLD | 0.49 |  |
| MDS | MDS-MLD | 0.18 |  |
| MDS | MDS-MLD | 0.00 |  |
| MDS | MDS-MLD, 5q- | 0.00 |  |
| MDS | MDS-EB-1, 5q- | 0.00 |  |
| MDS | MDS-EB-2 | 1.84 | 5-AC (Vidaza) |
| MDS | MDS-MLD | 0.00 |  |
| MDS | MDS-MLD | 3.42 |  |
| MDS | MDS-EB-1 | 5.05 | 5-AC (Vidaza) |
| MDS | MDS-EB-2 | 0.00 | 5-AC (Vidaza) |
| MDS | MDS-EB-2 | 11.46 | 5-AC (Vidaza) |
| MDS | MDS-EB-2 | 7.97 | 5-AC (Vidaza) |
| MDS | MDS-EB-2 | 12.66 |  |
| MDS | MDS-EB-2 | 14.21 |  |
| MDS | 5q- | 2.29 |  |
| MDS | MDS-EB-1 | 6.22 |  |
| MDS | MDS-EB-1 | 4.21 |  |
| MDS | MDS-RS | 1.21 |  |
| MDS | MDS-EB-2 | 0.69 |  |
| MDS | MDS | 19.68 |  |
| MDS | MDS-EB-2 | 1.69 |  |
| MDS | MDS | 24.32 |  |
|  |  |  |  |
|  |  |  |  |
|  |  |  |  |
|  |  |  |  |
|  |  |  |  |
|  |  |  |  |
|  |  |  |  |
| Hematological malignancies (HM) group (*n* = 19) | | |  |
| Disease State | Diagnosis | *SBSN* mRNA Fold change |  |
| HM | Mantle cell lymphoma | 0.01 |  |
| HM | T-cell lymphoma | 0.00 |  |
| HM | Diffuse large B-cell lymphoma | 0.00 |  |
| HM | Chronic lymphocytic leukemia | 0.05 |  |
| HM | Diffuse large B-cell lymphoma | 0.00 |  |
| HM | Hodgkin's lymphoma | 0.00 |  |
| HM | Hodgkin's lymphoma | 0.00 |  |
| HM | Mantle cell lymphoma | 0.00 |  |
| HM | Diffuse large B-cell lymphoma | 0.00 |  |
| HM | Follicular lymphoma | 0.00 |  |
| HM | Myeloproliferative neoplasms | 0.41 |  |
| HM | Diffuse large B-cell lymphoma | 0.00 |  |
| HM | Follicular lymphoma | 0.00 |  |
| HM | Chronic lymphocytic leukemia | 0.81 |  |
| HM | Follicular lymphoma | 0.31 |  |
| HM | Follicular lymphoma | 3.34 |  |
| HM | Myeloproliferative neoplasms | 0.00 |  |
| HM | Hodgkin's lymphoma | 0.27 |  |
| HM | Primary myelofibrosis | 0.14 |  |
|  |  |  |  |
|  |  |  |  |
|  |  |  |  |
| Healthy group (*n* = 8) | | |  |
| Disease State | Diagnosis | *SBSN* mRNA Fold change |  |
| Healthy | - | 1.665229 |  |
| Healthy | - | 1.368302 |  |
| Healthy | - | 0.8061129 |  |
| Healthy | - | 0 |  |
| Healthy | - | 0.3751661 |  |
| Healthy | - | 0.02528599 |  |
| Healthy | - | 0.006288377 |  |
| Healthy | - | 0.0797963 |  |
|  |  |  |  |
| Bone marrow mononuclear cells | | |  |
| Cohort #2 | | |  |
| MDS group (*n* = 48) | | |  |
| Disease State | Diagnosis | *SBSN* mRNA fold change |  |
| MDS | MDS-EB-2 | 2.08 |  |
| MDS | MDS-EB-2 | 5.30 |  |
| MDS | MDS-EB-1 | 1.66 |  |
| MDS | MDS-MLD | 0.84 |  |
| MDS | MDS-RS-SLD | 0.40 |  |
| MDS | MDS-RS-SLD | 1.08 |  |
| MDS | MDS-MLD | 0.72 |  |
| MDS | MDS | 1.11 |  |
| MDS | MDS-EB-2 | 2.01 |  |
| MDS | MDS-SLD | 0.59 |  |
| MDS | MDS-MLD | 1.47 |  |
| MDS | MDS-MLD | 1.59 |  |
| MDS | MDS | 2.60 |  |
| MDS | MDS-MLD | 0.75 |  |
| MDS | MDS-RS-SLD | 1.38 |  |
| MDS | MDS-EB-2 | 3.10 |  |
| MDS | MDS-MLD | 1.13 |  |
| MDS | MDS-RS-SLD | 1.15 |  |
| MDS | MDS | 2.99 |  |
| MDS | MDS-RS-SLD | 2.47 |  |
| MDS | MDS-EB-1 | 1.37 |  |
| MDS | MDS-SLD | 1.95 |  |
| MDS | MDS | 1.67 |  |
| MDS | MDS | 1.32 |  |
| MDS | MDS | 0.97 |  |
| MDS | MDS | 0.60 |  |
| MDS | MDS | 0.47 |  |
| MDS | MDS | 2.29 |  |
| MDS | MDS-MLD | 1.34 |  |
| MDS | MDS-EB-2 | 4.49 |  |
| MDS | MDS | 2.20 |  |
| MDS | MDS | 3.18 |  |
| MDS | MDS-EB-1 | 2.15 |  |
| MDS | MDS-MLD | 0.88 |  |
| MDS | 5q- | 2.28 |  |
| MDS | 5q- | 1.21 |  |
| MDS | 5q- | 1.90 |  |
| MDS | 5q- | 1.16 |  |
| MDS | 5q- | 1.32 |  |
| MDS | 5q- | 0.96 |  |
| MDS | 5q- | 1.17 |  |
| MDS | 5q- | 1.16 |  |
| MDS | 5q- | 2.19 |  |
| MDS | 5q- | 2.29 |  |
| MDS | 5q- | 2.19 |  |
| MDS | 5q- | 0.99 |  |
| MDS | 5q- | 2.37 |  |
| MDS | 5q- | 0.60 |  |
|  |  |  |  |
| Hematological malignancies (HM) group (*n* = 11) | | |  |
| Disease State | Diagnosis | *SBSN* mRNA fold change |  |
| HM | AML | 1.52 |  |
| HM | AML | 0.95 |  |
| HM | ALL | 1.33 |  |
| HM | AML | 1.71 |  |
| HM | CMML | 0.22 |  |
| HM | AML | 0.39 |  |
| HM | CML | 0.27 |  |
| HM | AML | 1.23 |  |
| HM | AML | 1.69 |  |
| HM | AML | 0.76 |  |
| HM | ALL | 1.09 |  |
