# Supplementary material for: Aberrantly elevated suprabasin in the bone marrow as a candidate biomarker of advanced disease state in myelodysplastic syndromes
Source: Mol Oncol. 2020 Aug 11;14(10):2403–19. doi: 10.1002/1878-0261.12768 (PMC7530796; doi:10.1002/1878-0261.12768)
Supplement: Supplementary file 3 — Table S2. Clinical characteristics of ‘MDS’ and ‘hematological malignancies’ patients groups of Cohort #3 including bone marrow blasts and T cells percentages, with bone marrow and peripheral blood SBSN concentrations. [file MOL2-14-2403-s003.docx]

| Clinical characteristics of patients and donors with blast and lymphocyte percentages, cytokine and SBSN levels detected in bone marrow (BM) plasma or peripheral blood (PB) plasma. Clinical characteristics of 'MDS' and 'hematological malignancies' patients groups of Cohort #3 including bone marrow blasts and T cells percentages, with bone marrow and peripheral blood SBSN concentrations. | | | | | | | | |
| --- | --- | --- | --- | --- | --- | --- | --- | --- |
| Cohort #3 | | | | | | | | |
| MDS Group (*n* = 42) | | | | | | | | |
| Sex | Diagnosis | IPSS | BM Blasts % | BM T lymphocytes % | BM SBSN pg/ml | PB SBSN pg/ml | Treatment | Treatment during sample acquisition |
| M | EB-2 | IPSS int II | 15.2 | 7.6 | 7868.3 | 460.5 | VIDAZA | no treatment |
| M | EB-2 | IPSS int II | 16.4 | 11.2 | 6155.5 | 698.7 | VIDAZA | VIDAZA |
| F | EB-2 | IPSS int II | 11.2 | N/A | 4967.3 | 813.8 | VIDAZA | no treatment |
| F | EB-2 | IPSS int II | 0.8 | 1.6 | 4199.2 | 451.2 | VIDAZA | VIDAZA |
| F | EB-2 | IPSS int I | 4.6 | 3.6 | 3847.8 |  | VIDAZA | no treatment |
| F | EB-2 | IPSS int II | 5.4 | 9.0 | 3460.6 | 484.6 | VIDAZA | VIDAZA |
| M | EB-2 | IPSS int II | 16.2 | 7.6 | 3335.3 |  | VIDAZA | no treatment |
| F | EB-2 | IPSS int II | 2.4 | 2.6 | 3145.0 |  | VIDAZA | no treatment |
| M | EB-2 | IPSS int II | 8 | 6.4 | 2966.4 |  | VIDAZA | VIDAZA |
| M | EB-2 | IPSS int II | 9.4 | 14.0 | 2802.8 |  | VIDAZA | VIDAZA |
| M | EB-2 | IPSS high | 12.8 | 5.6 | 2621.5 |  | VIDAZA | no treatment |
| F | EB-1 | IPSS int I | 1.6 | 36.8 | 2389.0 |  | VIDAZA | no treatment |
| M | EB-2 | IPSS high | 7 | 9.4 | 1832.8 |  | VIDAZA | VIDAZA |
| M | EB-2 | IPSS high | 11.6 | 32.0 | 1809.0 |  | VIDAZA | no treatment |
| M | EB-2 | IPSS int II | 2.4 | 12.6 | 1645.3 |  | VIDAZA | VIDAZA |
| F | EB-1 | IPSS int I | 19.2 | 14.2 | 1624.8 |  | VIDAZA | no treatment |
| F | EB-1 | IPSS low | 0.4 | 9.8 | 1276.0 | 0.0 | no treatment | no treatment |
| M | MLD | IPSS low | 0.7 | N/A | 897.8 |  | VIDAZA | VIDAZA |
| M | MLD | IPSS low | 0.4 | 19.2 | 858.1 | 0.0 | no treatment | no treatment |
| M | EB-1 | IPSS int I | 3.6 | 13.0 | 808.1 |  | no treatment | no treatment |
| M | t-MDS EB-2 | IPSS int II | 0.2 | 25.4 | 799.0 |  | VIDAZA | VIDAZA |
| F | EB-2 | IPSS int II | 9.2 | 10.8 | 730.7 | 0.0 | VIDAZA | no treatment |
| M | EB-1 | IPSS int I | 5.2 | 18.6 | 597.4 |  | VIDAZA | VIDAZA |
| M | EB-1 | IPSS int II | 6.4 | 34.4 | 556.9 | 167.6 | VIDAZA | VIDAZA |
| F | EB-2 | IPSS int II | 2.8 | 26.2 | 545.3 |  | VIDAZA | VIDAZA |
| M | t-MDS EB-2 | IPSS int II | 14.5 | 42.5 | 531.9 | 183.8 | VIDAZA | no treatment |
| M | EB-2 | IPSS int II | 7 | 11.2 | 515.3 |  | VIDAZA | no treatment |
| F | EB-2 | IPSS high | 10.6 | 12.6 | 451.5 |  | VIDAZA | VIDAZA |
| M | EB-2 | IPSS high | 10 | 8.0 | 405.3 |  | VIDAZA | VIDAZA |
| F | EB-2 | IPSS int II | 13 | 12.2 | 397.8 |  | VIDAZA | VIDAZA |
| F | MLD | IPSS int I | 0.8 | 33.6 | 340.2 |  | no treatment | no treatment |
| M | EB-1 | IPSS int I | 7.2 | 18.2 | 318.8 |  | VIDAZA | no treatment |
| F | EB-1 | IPSS int II | 4 | 7.2 | 290.2 |  | VIDAZA | no treatment |
| F | EB-2 | IPSS int II | 13.2 | 29.2 | 239.0 |  | VIDAZA | no treatment |
| M | MLD | IPSS low | 2 | 4.0 | 236.5 |  | VIDAZA | VIDAZA |
| F | EB-1 | IPSS int II | 3 | 15.6 | 171.5 |  | VIDAZA | VIDAZA |
| M | EB-2 | IPSS int II | 14.4 | 4.4 | 137.9 |  | VIDAZA | VIDAZA |
| F | EB-2 | IPSS high |  |  | 5092.0 |  | VIDAZA | VIDAZA |
| M | MLD | IPSS int II |  |  | 4526.0 |  | VIDAZA | VIDAZA |
| F | EB-2 | IPSS int II |  |  | 13381.5 |  | no treatment | no treatment |
| F | EB-2 | IPSS high |  |  | 0.0 |  | VIDAZA | no treatment |
| M | EB-2 | IPSS int II |  |  | 405.0 | 327.0 | VIDAZA | VIDAZA |

| MDS 5q- Group (*n* = 12) | | | | | |
| --- | --- | --- | --- | --- | --- |
| Sex | Diagnosis | IPSS | BM Blasts % | BM T lymphocytes % | BM SBSN pg/ml |
| M | MLD | IPSS int I | 5.6 | 14.2 | 746.2 |
| F | EB-1 | IPSS low | 2.6 | 14.6 | 717.6 |
| F | MLD | IPSS int I | 1.6 | 8.8 | 633.1 |
| M | EB-1 | IPSS int I | 7 | 16.2 | 487.8 |
| F | EB-1 | IPSS int II | 6 | 11.4 | 408.1 |
| F | MLD | IPSS int II | 0.4 | 23.6 | 401.5 |
| M | EB-1 | IPSS int I | 3.8 | 25.6 | 300.3 |
| F | MLD | IPSS int I | 4 | 36 | 226.5 |
| M | EB-2 | IPSS high | 14 | 42.4 | 128.3 |
| M | EB-1 | IPSS int I | 3.2 | 24 | 42.7 |
| F | MLD | IPSS low | 3.6 | 50.4 | 5.7 |
| F |  | IPSS low |  |  | 55 |

| AML group (*n* = 7) | | | | | | |
| --- | --- | --- | --- | --- | --- | --- |
| Sex | Diagnosis | IPSS | BM Blasts % | BM T lymphocytes % | | BM SBSN pg/ml |
| M | AML | IPSS high | 35.4 | 13.0 | 1167.0 | |
| M | AML (5q-) | IPSS high | 48.8 | 9.6 | 712.9 | |
| M | AML | IPSS high | 22.4 | 8.0 | 456.9 | |
| M | AML | IPSS int I | 27.6 | 18.0 | 218.8 | |
| M | AML | IPSS high | 21 | 20.0 | 115.2 | |
| M | AML | IPSS high | 25.2 | 20.6 | 26.7 | |
| F | AML | IPSS high | 25 | 38.6 | 97.3 | |

| Patients of MDS group in MDS state and MDS-transformed AML state (*n* = 3) | | | | | |
| --- | --- | --- | --- | --- | --- |
|  |  |  |  |  |  |
| Sex | Diagnosis | IPSS | BM Blasts % | BM T lymphocytes % | BM SBSN pg/ml |
| F | EB-2 | IPSS int II | 5.4 | 9 | 3460.6 |
| F | AML | IPSS int II | 24.2 | 11.2 | 1512.9 |
| F | EB-2 | IPSS int II | 0.8 | 1.6 | 4199.2 |
| F | AML | IPSS int II | 49.2 | 3.4 | 1175.3 |
| F | EB-1 | IPSS int I | 19.2 | 14.2 | 1624.8 |
| F | AML | IPSS int II | 22 | 17.8 | 112.8 |

| non-MDS (*n* = 12) | | |
| --- | --- | --- |
| Sex | Diagnosis | BM SBSN pg/ml |
| F | healthy | 27.75 |
| F | healthy | 969 |
| M | healthy | 307 |
| M | healthy | 291.5 |
| M | trombocytopenia | 450.25 |
| M | trombocytopenia | 42.75 |
| M | trombocytopenia | 409 |
| F | trombocytopenia | 955 |
| M | Chronic anemia | 50 |
| F | Multiple myeloma | 20 |
| M | T lymphoproliferation | 89.9 |
| M | Multiple myeloma | 58 |
