# Supplementary material for: Aberrantly elevated suprabasin in the bone marrow as a candidate biomarker of advanced disease state in myelodysplastic syndromes
Source: Mol Oncol. 2020 Aug 11;14(10):2403–19. doi: 10.1002/1878-0261.12768 (PMC7530796; doi:10.1002/1878-0261.12768)
Supplement: Supplementary file 4 [file MOL2-14-2403-s004.docx]

**Supplementary legend**

**Supplementary Figure 1.** Immunofluorescence detection of SBSN (green) using anti-SBSN antibody and Alexa Fluor 647 goat anti-rabbit in human glioblastoma U373 cells irradiated with a single dose of 2 Gy 48 hours after siSBSN or siNC transfection (**A**). Nuclei were stained with DAPI (blue), with *n* = 3 per group, Scale bar, 10 µm. (**B**) Immunohistochemistry of SBSN in MDS bone marrow smears using anti-SBSN antibody and IgG-HRP goat anti-rabbit. Representation of the SBSN-negative population of cell in bone marrow smears of MDS patients (EB-1 and EB-2; *n* = 3, scale bar, 10 µm). (**C**) Correlation between log2 BM SBSN protein levels (pg/mL) and log2 BM B lymphocyte percentage of MDS, MDS 5q-, and AML patients (*n* = 53). (**D**) Correlation between log2 BM T and B lymphocyte percentage of MDS, MDS 5q-, and AML patients (*n* = 47). (**E**) Correlation between log2 BM CCL2 protein levels (pg/mL) and log2 BM T lymphocyte percentage of MDS, MDS 5q-, and AML patients (*n* = 51). (**F**) Correlation between log2 BM CCL2 protein levels (pg/mL) and log2 BM B lymphocyte percentage of MDS, MDS 5q-, and AML patients (*n* = 45).
