# Supplementary material for: Aberrantly elevated suprabasin in the bone marrow as a candidate biomarker of advanced disease state in myelodysplastic syndromes
Source: Mol Oncol. 2020 Aug 11;14(10):2403–19. doi: 10.1002/1878-0261.12768 (PMC7530796; doi:10.1002/1878-0261.12768)
Supplement: Supplementary file 5 [file MOL2-14-2403-s005.docx]

**Supplementary tables**

**Supplementary Table S1** Disease state, diagnosis and bone marrow *SBSN* mRNA fold change of MDS patients, or 'hematological malignancies' (HM) patients, and healthy donors. Fold change of mRNA was normalized to HM patients' group.

**Supplementary Table S2** Patients' groups (MDS, MDS 5q- and AML) and their diagnosis including sex, The WHO and IPSS classification, SBSN protein levels, blasts and T lymphocytes occurrence and non-MDS patients with SBSN protein levels.
